# Soil C, N, P and Its Stratification Ratio Affected by Artificial Vegetation in Subsoil, Loess Plateau China

**DOI:** 10.1371/journal.pone.0151446

**Published:** 2016-03-17

**Authors:** Jian Deng, Pingsheng Sun, Fazhu Zhao, Xinhui Han, Gaihe Yang, Yongzhong Feng, Guangxin Ren

**Affiliations:** 1 College of Agronomy, Northwest A&F University, Yangling, Shaanxi, China; 2 The Research Center of Recycle Agricultural Engineering and Technology of Shaanxi Province, Yangling, Shaanxi, China; Institute for Sustainable Plant Protection, C.N.R., ITALY

## Abstract

Artificial vegetation restoration can induce variations in accumulation and distribution of soil carbon (C), nitrogen (N) and phosphorus (P). However, little is known about variations in soil C, N and P nutrient fraction stratification following artificial vegetation in Loess Plateau China. Based on the hypothesis that re-vegetated can improve soil quality and stratification ratios (SR) can be used as an indicator to evaluate soil quality. This study measured contents and storages of soil organic carbon (SOC), total nitrogen (TN), total phosphorus (TP) and their SRs in topsoil (0–20 cm) and subsoil (20–60 cm) in three 30-year re-vegetated lands that had been converted from arable land (*Robinia pseudoacacia L*., *Caragana Korshinskii Kom*. and abandoned cropland with low interferences and few management measures) and one slope cropland (SC) as a control for three soil profiles(0–20 cm, 20–40 cm and 40–60 cm) from June 2009 to June 2013. The results showed that the contents and storages of SOC, TN and TP in re-vegetated land were significantly higher than those in the SC in both topsoil and subsoil. The storages of SOC, TN and TP in the topsoil (0–20 cm) of the re-vegetated lands increased by 16.2%-26.4%, 12.7%-28.4% and 16.5%-20.9%, respectively, and increased by smaller but significant amounts in subsoil from 2009 to 2013. The SRs for SOC, TN and TP in the re-vegetated lands were mostly >2 (either for 0–20:20–40 cm or 0–20:40–60 cm) and greater than that in the SC. The SRs showed an increasing trend with increasing restoration age. The results also showed that the land use type and soil depth were the most influential factors for the SRs and storages, and the SRs of SOC and TN had significantly positive correlations with their storages. The SRs were concluded to be a good indicator for evaluating the soil quality, which can be significantly enhanced through vegetation restoration. Moreover, vegetation restoration can significantly enhance SOC, TN and TP accumulation in both topsoil and subsoil.

## Introduction

Artificial vegetation restoration, the conversion of non-vegetated or arable land to a plant covered land, has attracted increasing social attention and has become one of the hottest topics of ecological study as an efficient measure to repair destroyed natural ecosystems [1,2]. In addition to altering the understory diversity of species[3], artificial vegetation has substantial effects on the accumulation and distribution of soil nutrient components, i.e., soil carbon (C), nitrogen (N) and phosphorus (P) [3]. For instance, Zhao et al. [4] and Fu et al. [5] illustrated that both large-scale artificial forestry and grasslands that were based on cropland significantly increased soil organic carbon (SOC), soil total nitrogen (TN) and soil total phosphorus (TP) sequestration. Korkanç[6] showed that the SOC in the 0–20 cm increased from 0.5% to 1.3% 15 years after planting black pine on bare land. Lima et al.’s[7] study obtained similar results in eucalyptus plantations and further revealed that SOC accumulation has strong time dependence. Evaluating variations in soil C, N and P contents and soil quality, along with re-vegetation types and years, is very important because the soil nutrients are the foundation of complex biochemical process and essential to the survival of bio-organisms [8]. In addition, compared to the long history of surface soil studies (0–20 cm or shallower), subsoil (sub-surface soil at 20–60 cm) has only been the subject of intensive research in recent years because subsoil has since been recognized to contribute greatly to C, N and P accumulation. Amundson[9] reported that more than 50% of the global soil C was stored in the subsoil (20–100 cm layer). Guo and Gifford’s [10] results showed that at least 61% of the total soil C was stored below 30 cm in the northern circumpolar permafrost region, and other research has shown subsoil plays an even more important role as a CO_2_ sink than top soil [11,12]. Zhang et al. [13] declared that subsoil greatly interplays with ground vegetation, and more than 60% of the vegetation roots in the Loess Plateau were distributed in the 20–60 cm soil layer. Therefore, understanding the effect of long-term vegetation recovery on changes in SOC, TN and TP in subsoil, which is not well documented, is important.

The stratification of a soil’s properties and compositions, especially soil C, N and P, is very common in natural and artificial vegetation and cropland [14–16]. The stratification ratio (SR) is defined as the ratio of a soil property at the surface layer to that at a deeper layer. High SR values (usually >2) indicate good soil quality [16]. The SR is widely used to estimate cropland soil as an indicator of the soil quality. For instance, the SR of SOC at depths of 0 to 5 cm and 12.5 to 20 cm was reported to range from 1.1 to 1.9 under conventional tillage and from 2.1 to 3.4 under no tillage [16]. The SRs of soil nutrients and other properties were also used to assess crop soil quality under different cover crop managements and tillage methods [17,18]. In recent years, the SR was applied to natural ecosystems and artificially vegetated land as an indicator to evaluate the soil condition. Francaviglia et al.’s [19]study showed that the SRs of SOC and TN under long-term artificial cork oak forest were >4, larger than those of vineyards, hay cropland and abandoned vineyards. A study from Zhao et al. [20] assessed the SRs of SOC and TN in three typical conversion lands in the Loess Hilly Region of China and illuminated that using SR as an indicator of soil quality in re-vegetated land was proper. Nevertheless, similar studies are still limited, and evidence for using SRs to estimate soil quality in the subsoil of artificially vegetated land should be supplemented.

The Loess Plateau of China (LPC), which has an area of 62.4×10^4^ km^2^, is the most vulnerable ecological environment in the world [21]. Approximately 72.3% of the total area is eroded, and the average erosion rate has reached 150 Mg/ha per year before governance [5]. To control soil erosion and restore vegetation, the Chinese government initiated the “Grain-to-Green Program” (GTGP) in 1999 to convert steep cultivated land (with a slope of over 25°) into forests (trees and shrubs) or grasslands [22]. Although the original aim was to prevent soil and water erosion, vegetation recovery also considerably affects soil element circulation and soil quality improvement in the LPC. Previous studies estimated the effects of re-vegetation based on carbon sequestration [4], soil stoichiometry proportions (C:N:P) [23], soil labile organic matter and carbon management indexes [24] and soil aggregates [25] in the LPC. However, information regarding the assessment of soil quality restoration along with re-vegetation using SRs in the LPC is still scarce, especially research on subsoil.

Therefore, this study aimed to verify the following hypotheses: (1) converting cropland to artificial vegetation can improve soil fertility attributes in topsoil and subsoil, (2) SR can be used as an indicator to evaluate the soil quality in artificially vegetated land, and (3) re-vegetation can improve the soil quality in the LPC.

## Materials and Methods

We declare that the study area required no specific permissions and the location was open public domain and not protected in any way. We confirm that the field studies do not involve endangered or protected species. Land use history information was obtained from interviews with local farmers(Mr. Xueqing Zhao, forest ranger of Zhenwudong Town in Ansai county, Shaanxi province, China), experimental station managers (Mr. Yibin Zhang, Soil and Water Conservation Experiment Station, Northwest A&F University, Ansai County, Shaanxi Province, China) and records of local forestry administrative department.

### Research area

**Fig 1:**
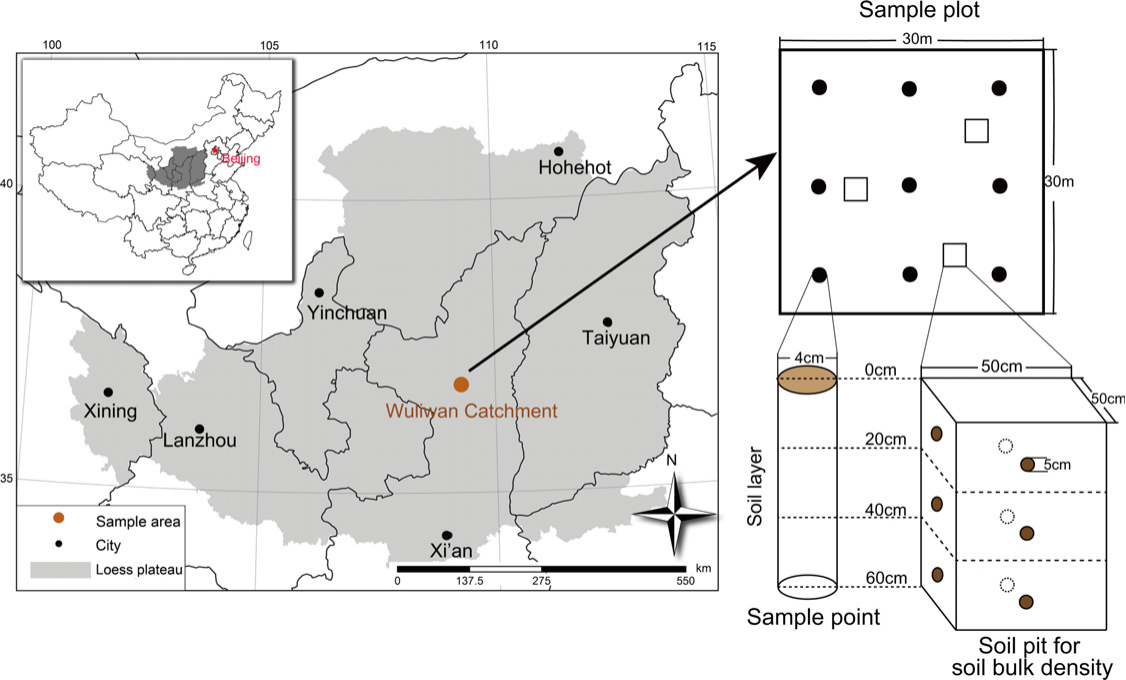
Location of the Wuliwan catchment in Loess Plateau of China and the sampling process. The map was plotted by Arcgis9.3.

This study was established in the Wuliwan catchment of Ansai County, northern Shaanxi, China (36°46′42″~36°46′28″N, 109°13′46″~109°16′03″E), which is located in the central region of the LPC (Fig 1). This area has a typical semi-arid climate with an annual average temperature of 8.8°C and an average annual precipitation of 505 mm. Approximately 60% of the precipitation occurs between July and September, and the precipitation varies greatly in different years. The landform of the study area is a typical loess hilly landscape with an average elevation of 1250 m above sea level. The soil in this region is Calciustepts soil that developed from wind-accumulated loess. The sand (2–0.05 mm) and silt (0.05–0.002 mm) contents account for 29.2% and 63.6%, respectively, in the 0–20 cm soil layer [26]. The soil in this area has a weak resistance to erosion, with an extremely high erosion modulus of 10,000 to 12,000 Mg•km^−2^•yr^−1^ before the restoration efforts began [26]. Arable farming mostly occurs on sloped lands without irrigation, and natural vegetation was destroyed by cultivation before the 1970s. The forestland area increased significantly after approximately 30 years of artificial vegetation restoration.

**Fig 2:**
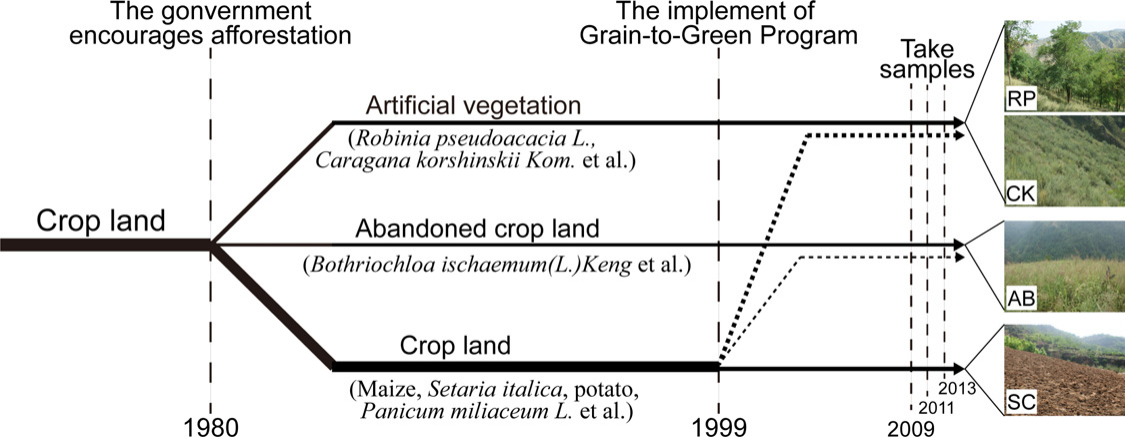
Chronology and land use history of the sample site. RP, CK, AB and SC represent *Robinia pseudoacacia L*. land, *Caragana korshinskii Kom*. Land, abandoned cropland and slop cropland, respectively, same in the rest figures.

The major agricultural land use type in the study area is slope cropland (SC), mainly plant maize (*Zea mays L*.), foxtail millet (*Setaria italica*) and broomcorn millet (*Panicum miliaceum L*.). Crop farming depends on the rainfall and manure is main fertilizer. As the chronology of sample site shown in Fig 2, from 1980, the government encourages people to plant trees and reforestation to improve the environment. So a part of SC was replanted with forest and shrubs to reduce soil erosion with major tree species of *Robinia pseudoacacia L*. (RP) and *Caragana* korshinskii *Kom*.(CK). During the same period, abandoned cropland (AB) was also generated, due to its extremely low productivity and long distance from farmers’ residences [27]. In 1999 with the implementation of GTGP, another more SC lands were converted to abandoned cropland and reforestation land.

### Soil sampling

Four land use types in the Wuliwan catchment, including three re-vegetation types (i.e., 30-year-old RP land, CK land and AB, generated from 1980) and SC, were selected in September 2008 based on the land use history (Table 1). Three 30 m×30 m plots were randomly selected in each land use type for follow-up observations and sampling. The distances of the three plots for each type was within 1.5 km to ensure consistent of climatic and other conditions. All the sites were topographically similar (i.e., slope aspect, slope degree etc.) with the same elevation.

**Table 1:** General situation of the sample plots.

Plots Type	Location	Elevation (m)	Slope Aspect (°)	Slope Degree (°)	Age (a)	Litter biomass (g/m^2^, in 2009)	Bulk density (0–20cm)(g/cm^3^)	Canopy Density (%)	Undergrowth dominant species (confirming by importance value)
RP-1	36°51.637’ N, 109°21.036’E	1207	NbyW22	30	30	1302.12	1.12	75	*Artemisia gmelini; Bidens pilosa*
RP-2	36°51.618’ N, 109°20.994’ E	1210	NbyW25	30	30	1001.39	1.09	75	*Artemisia gmelini; Bidens pilosa*
RP-3	36°51.596’ N, 109°20.950’ E	1256	NbyW25	35	30	1256.21	1.05	65	*Artemisia gmelini; Bidens pilosa*
CK-1	36°51.592’ N, 109°21.057’ E	1247	NbyW10	30	30	885.63	1.06	85	*Heteropappus altaicus (Willd) Novopokr; Stipa bungeana Trin*.
CK-2	36°51.941’ N, 109°21.027’ E	1244	NbyW12	35	30	806.24	1.06	85	*Heteropappus altaicus (Willd) Novopokr; Stipa bungeana Trin*.
CK-3	36°51.905’ N, 109°20.984’ E	1311	NbyE10	35	30	785.68	1.05	90	*Heteropappus altaicus (Willd) Novopokr; Stipa bungeana Trin*.
AB-1	36°51.814’ N, 109°21.043’ E	1224	NbyE60	40	30	692.35	1.11	75	*Bothriochloa ischaemum(L*.*)Keng*
AB-2	36°51.778’ N, 109°21.053’ E	1224	NbyE60	37	30	665.88	1.11	80	*Bothriochloa ischaemum(L*.*)Keng*
AB-3	36°51.740’ N, 109°21.089’ E	1223	NbyE65	35	30	785.68	1.09	80	*Bothriochloa ischaemum(L*.*)Keng*
SC-1	36°51.906’ N, 109°21.073’ E	1246	SbyE40	18	>30	—	1.10	—	—
SC-2	36°52.134’ N, 109°20.882’E	1258	SbyW50	10	>30	—	1.12	—	—
SC-3	36°52.153’ N, 109°20.907’E	1273	SbyW50	25	>30	—	1.13	—	—

Soil samples were collected in mid-June of 2009, 2011 and 2013for laboratory analyses. Nine sample points with an “S” shape were selected in each plot (Fig 1). Soil samples were collected at each point from three soil layers, namely, 0–20 cm, 20–40 cm and 40–60 cm, by using a soil auger (4 cm in diameter). The soil samples in each plot from the 9 sample points at the same depth were adequately mixed and reduced by coning quartering to appropriate quantities. Then, the soil samples were stored in well-ventilated bags for laboratory analyses. All the samples were collected at least 80 cm from trees after removing the litter layer to avoid impact of root and litters.

The soil bulk density(ρ) was determined with soil cores method[28]. Soil bulk density samples were collected randomly from three points in each plot (Fig 1). Pits (50 cm×50 cm) were opened at each point for sample collection. Three cores were obtained from the middle portions of the corresponding soil layer(0–20 cm, 20–40 cm and 40–60 cm) by using a core sampler with of 5 cm×5 cm steel cylinders. The steel cylinders with the samples were dried in an oven at 104°C for 48 h, and ρ was calculated as the weight: volume ratio (g/cm)[29].

### Laboratory analyses

Soil samples were sieved through a 2 mm griddle and then air-dried and stored at room temperature (25–28°C) to determine the soil chemical properties. The soil organic carbon content was determined by using the K_2_Cr_2_O_7_ oxidation method based on Walkley-Black’s method[30]. The soil TN and TP contents were determined by using the Kjeldhal method and Mo-Sb anti-spectrophotography method, respectively [31]. However, the Walkley-Black method usually underestimates the SOC values compared to dry combustion methods [32], so we transformed the K_2_Cr_2_O_7_ oxidation method results into SOC values by using the Correction Factor(CF) in [33] with the following equation:

$$SOC={SOC}_{WB}\times CF$$

where SOC is the corrected soil organic carbon content value, SOC_WB_ is the soil organic carbon content that was determined by using the K_2_Cr_2_O_7_ oxidation method, and CF is the correction factor cited from Tivet et al.’s[33] study for different land use types. We use the average correction factor for each layer and land use type as presented in [33](Table 2).

**Table 2:** Correction factors for SOC analysis.

Land use types	Types in reference	0–20cm	20–40cm	40–60cm
RP	Forest	1.49	1.57	1.37
CK	Forest	1.49	1.57	1.37
AB	Native grassland	1.47	1.55	1.37
SC	Conventional tillage	1.44	1.46	1.40

The SOC, TN and TP storages were calculated as follows:

$$Storages\left(SOC,TN\;or\;TP\right)=C_{SOC,TN\;or\;TP}\;\times \rho \times H_{e}\times 10^{-1}$$

where Storages(SOC, TN or TP) is the storage of SOC, TN or TP (Mg·ha^−1^) and C_SOC,TN or TP_ is the content (g·kg^−1^) of SOC, TN or TP. ρ represents the bulk density (g·cm^−3^)and H_e_ (cm) is the equivalent soil thickness. The SOC, TN and TP storages were calculated by using the equivalent soil mass theory to revise the differences in bulk density among treatments[34]. Thus, H_e_ was calculated by using the following equation:

$$H_{e}=20+{H\;}_{\text{add}}=20+(\frac{M_{\text{soil}\;v}{-M}_{soil\;SC}}{\rho_{soil\;SC}})$$

where H_e_ is the equivalent soil thickness, H_add_ is the additional soil thickness that is required to attain the equivalent soil mass; M _soil v_ is the mass of soil in the respective soil layers in the vegetation land, M _soil SC_ the mass of the soil in the corresponding soil layers in the SC land, ρ_soil SC_ is the bulk density of the SC in the corresponding layer, and the value 20 is the actual soil depth in each layer.

The SR values of SOC, TN and TP were calculated by using the surface content in the layer of 0–20 cm layer divided by the corresponding content at a deeper layer (20–40 cm or 40–60 cm). For example, the SR of the SOC contents at 0–20:20–40 was calculated by using the SOC content in the 0–20 cm layer divided by that in the 20–40 cm layer.

### Statistical analysis

The Kolmogorov-Smirnov method was used to test normal distributions; all the data were distributed normally (P>0.05 for each null hypothesis). We use an unbalanced three-way ANOVA with fixed treatment effects of land use types, years, and soil depth to test these treatment effects on contents and storages of SOC, TN, TP and SRs; detailed ANOVA results tables are listed in the Tables in the S1 Table. Comparisons among the treatment means were made by using Duncan's multiple range test calculated at 5%. Differences of P<0.05 were considered statistically significant. Correlations between the SR values and storages of SOC, TN and TP were estimated by using Pearson linear correlation coefficients analysis. The statistical procedures were conducted with the software program SAS (SAS Institute Inc., North Carolina, USA).

Principal Component Analysis (PCA) was performed to show the differentiation between samples and to discriminate which treatments (land use types, years and soil depth) may have greater influence on several variables (SRs and storages of SOC, TN and TP). The PCA was conducted with Canoco (version 5.0. Microcomputer Power, Ithaca, USA)[35]. The data were standardized and centralized to account for the different magnitudes of the parameters and indicators to contribute to the principal component calculation.

Analysis of Similarities (ANOSIM) was performed to show a quantized and clearer effect of treatments on each variable after PCA. The sample statistic R was proposed to measure the differences between groups; details of this calculation and theory can be found in [36]. In this study, a significance level below 0.01 was considered as there are significant differences existed among the groups. For each indicator, all the samples were respectively grouped based on each treatment (land use types, years and soil depth), and greater R-value indicate better separating capacity of corresponding treatment when statistically significant; more specifically, the treatment had a greater effect on the variables. The ANOSIM was performed with the PRIMER” (v7.0) package[37].

## Results

### Contents of SOC, TN, and TP

**Fig 3:**
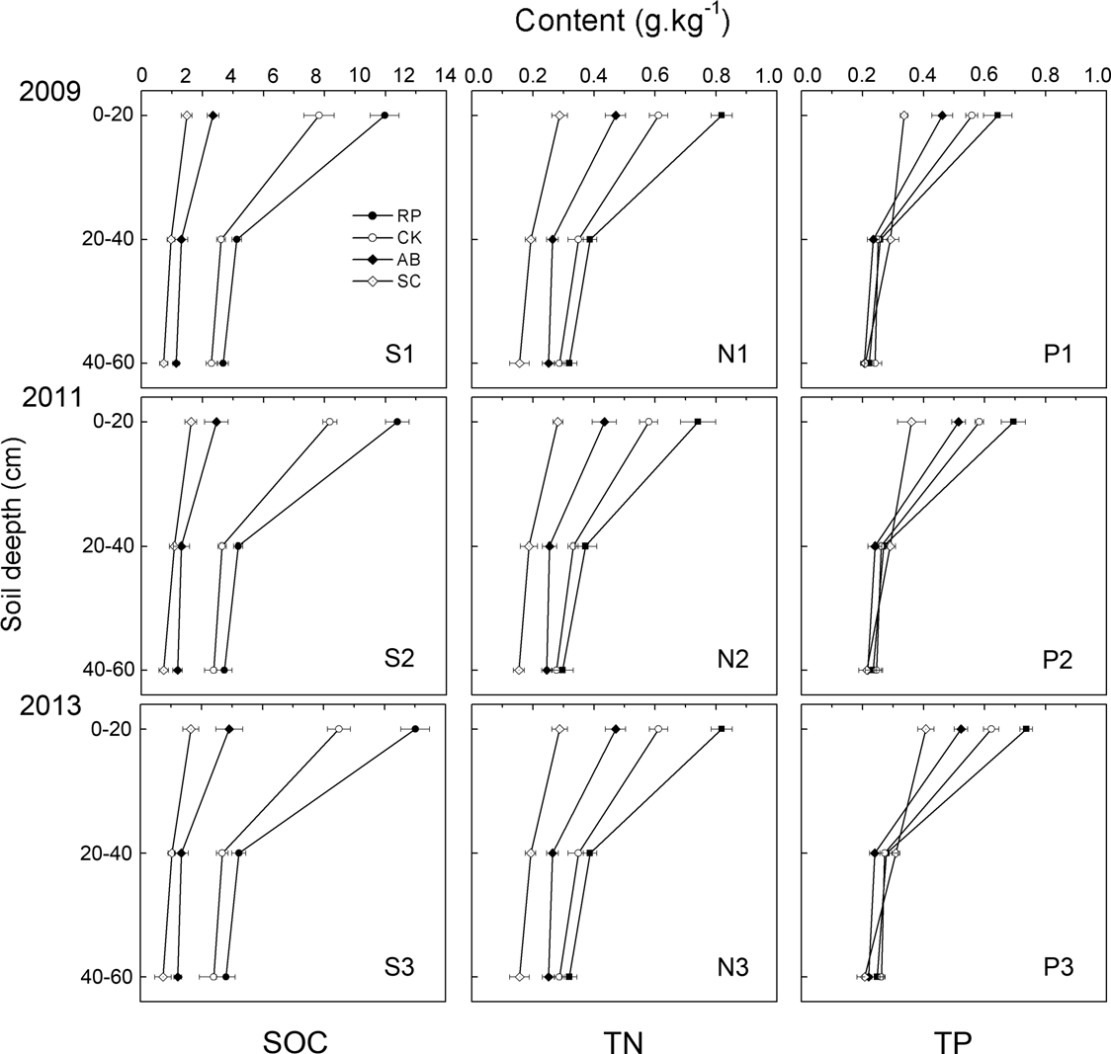
Soil contents of SOC, TN, and TP for different land use types and soil depth in 2009, 2011, and 2013. Bars represent standard errors.

The land use types (RP, CK, AB and SC) and years of vegetation restoration significantly affect the soil SOC, TN and TP contents (P<0.05), as shown in Fig 3. The SOC, TN and TP contents decreased with increasing soil depth under each land use type. In 2013, the SOC, TN and TP contents in the surface soil (0–20 cm) of re-vegetated lands (RP, CK and AB) were 80.4%-466.0%, 63.9%-184.7% and 28.3%-80.9% larger than those of the SC, respectively (P<0.05). Meanwhile, the SOC and TN contents in the subsurface soil (20–40 cm and 40–60 cm) of the re-vegetated lands were also significantly higher than those of the SC (P<0.01), while the TP content exhibited no significant differences. The variances in the surface soil were significantly higher than those in the subsurface soil (P<0.01). Moreover, the SOC, TN and TP contents decreased by 60.9%-71.4%, 45.7%-61.0%, 49.2%-66.3% when the soil depth changed from 0–20 cm to 40–60 cm among the different re-vegetated lands (P<0.05). Among all the re-vegetation types, RP offered greater SOC, TN and TP increments than the others in each soil depth (P<0.01) compared to the SC. The SOC, TN and TP contents in different soil depths and land use types were uniformly distributed for 2009, 2011 and 2013.

From 2009 to 2013, the soil SOC, TN and TP contents in the three re-vegetation types at depths of 0–20 cm increased by 13.0%-24.0%, 9.7%-20.8% and 11.4%-14.5%, respectively (Fig 3). Meanwhile, the SOC, TN and TP contents increased by 2.3%-3.0%, 4.8%-10.0%, and 2.0%-8.6% at 20–40 cm, respectively, and 4.3%-7.1%, 8.1%-14.9%, and 6.6%-11.8% at 40–60 cm, respectively. The largest increment was found in the topsoil and decreased as the soil depth increased. This result suggests that the soil elements in each profile had obvious properties of surface-aggregation, which may be caused by the strong interference and interchange of materials in the surface soil layer. The SOC, TN and TP contents of the SC also showed a significant increase in the topsoil (P<0.05) from 2009 to 2013, but not in the subsoil. In addition, the variations in the SOC, TN and TP tended to be more salient in RP and CK compared to AB and SC.

### Changes in SOC, TN, and TP storages

**Fig 4:**
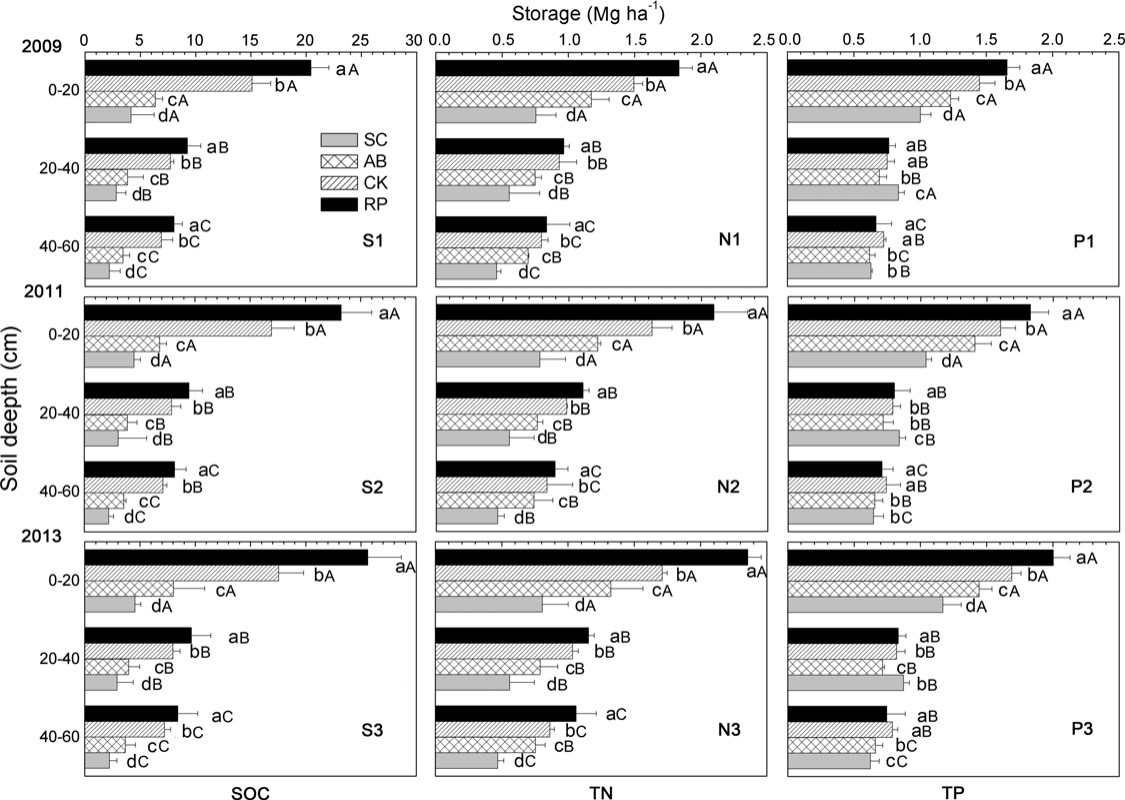
Soil storage of SOC, TN, and TP of different land use types and soil depth in 2009, 2011, and 2013. Columns with different capital letters indicate significantly difference among different soil depth in same land use types; columns with different small letters indicate significantly different of among different land use types in same soil depth. All significances are been tested at P<0.05 level of probability. Error bars represent standard errors.

The soil SOC, TN and TP storages were correlated with the land use types, soil depths and restoration years (Fig 4). In 2013, the SOC, TN, and TP storages in the 0–20 cm soil layer in the re-vegetated land types were 8.1–25.6 Mg.ha^-1^, 1.3–2.4 Mg.ha^-1^ and 1.4–2.0 Mg.ha^-1^, which were significantly higher than those in the SC (P<0.01). The SOC and TN storages of the re-vegetated lands also significantly increased at depth of 20–40 cm and 40–60 cm compared to those in the SC (P<0.01), but the storage and the increment decreased as the soil depth increased (P<0.05). Although the TP storage in the subsoil was significantly lower than that in the topsoil (P<0.01), not all the differences were significant between 20–40 cm and 40–60 cm for the same land use type and between different land use types at the same soil depth. Additionally, significant differences in the SOC and TN storage in each soil depth range and the TP storage in the 0–20 cm layer were observed among RP, CK and AB. The storages of the different land use types followed the order of RP>CK>AB>SC (P<0.01). The soil from 20 cm to 60 cm stored 41.3%-63.1% of the SOC, 48.5%-56.1% of the TN and 44.1%-56.1% of the TP from 0–60 cm soil profile. The biggest proportions were found in the SC. The distributions of the SOC, TN and TP storages for different soil depths and land use types were very similar in 2009, 2011 and 2013.

The soil SOC, TN and TP storages of the three vegetation restoration types changed significantly from 2009 to 2013 (P<0.01) (Fig 4), especially in the 0–20 cm soil layer, where thry increased by 16.2%-26.4%, 12.7%-28.4% and 16.5%-20.9%, respectively. From 2009 to 2013, the SOC, TN and TP storages increased by 3.4%-4.1%, 5.5%-19.6%, and 3.0%-9.1% in the 20–40 cm layer and by 4.3%-7.1%, 8.1%-26.9%, and 6.6%-11.8% in 40–60 cm layer, respectively. However, the SOC, TN and TP storages of the SC showed slight increases in the 0–20 cm soil layer, and no significant changes were observed in the 20–40 cm or 40–60 cm layer (P<0.05). RP represented the highest potential for SOC, TN and TP sequestration out of all the land use types, followed by CK.

**Fig 5:**
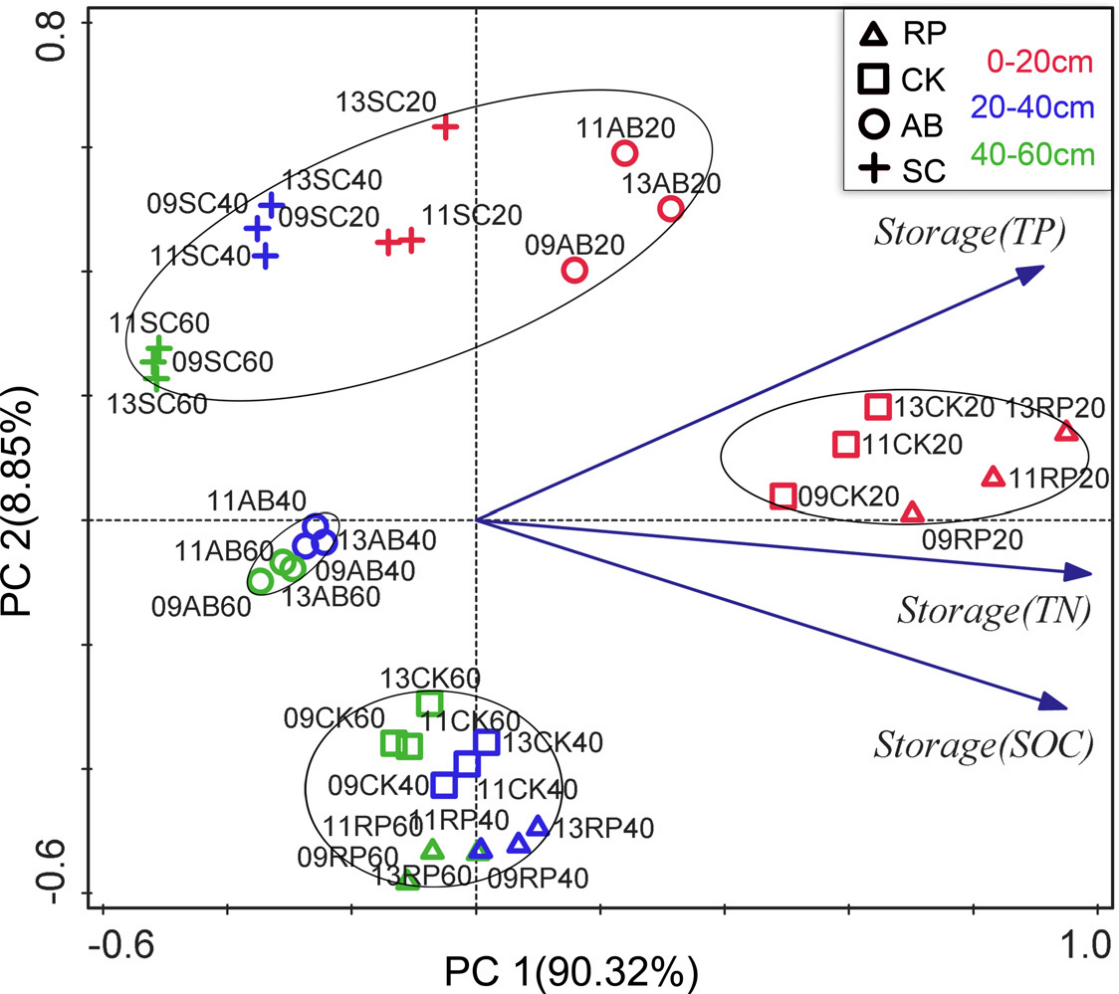
Principal component analysis (PCA) loadings for the soil SOC, TN and TP storage samples. Symbols with different shape represent different land use types: triangle is RP, square is CK, roundness is AB and cross is SC. Symbol with different color represent different soil depth: red for 0–20cm, blue for 20–40cm and green for 40–60cm. The ellipse is the approximate group of samples.

PCA was performed to discriminate the effects of land use types, soil depth and years of vegetation restoration on the SOC, TN and TP storages. The first two principal components successfully differentiated the samples, as shown in Fig 5. The cumulative variance contribution of PC1 (90.3%) and PC2 (8.9%) was 99.2%. In the PCA factor loading plots (Fig 4), the samples from the 0–20c m soil layer are grouped in the top-right corner of the coordinate system, with the 20–40 cm and 40–60 cm plots in the lower-left corner. The samples are also grouped by their land use types, i.e., sample plots of AB and SC are in the top-left comer and RP and CK plots are in the lower-right corner. The years did not group the sample plots in the scores plot. The land use types and soil depth evidently have stronger resolving power to distinguish variations in the SOC, TN and TP storages.

The R-values for ANOSIM showed that the SOC, TN and TP storages were all dissimilar from the groups that were derived from each treatment (P<0.01) (Table 3). The most influential factor for the SOC and TN storages was the land use type (R = 0.997 and 0.972, P<0.01), while that for the TP storage was soil depth (R = 0.929, P<0.01). Taken together, the land use type was the most influential factor for the SOC, TN and TP storages, with soil depth the second-most influential factor. The restoration years had a significant R value (greater than 0.8), although this value was the smallest.

**Table 3:** ANOSIM R-values of SOC, TN and TP storages^a^.

	Storage^b^	Storage(SOC)	Storage(TN)	Storage(TP)
**Land use type**	0.997	0.997	0.972	0.745
**Year**	0.801	0.858	0.714	0.640
**Soil depth**	0.983	0.993	0.968	0.929

^a^The R-values are the average value of pairwise tests for each treatment and are all statistically significant.

^b^The R-values for “Storage” are from comprehensive analyses of each treatment for the SOC, TN and TP storages.

### Stratification ratios of the SOC, TN and TP content

**Fig 6:**
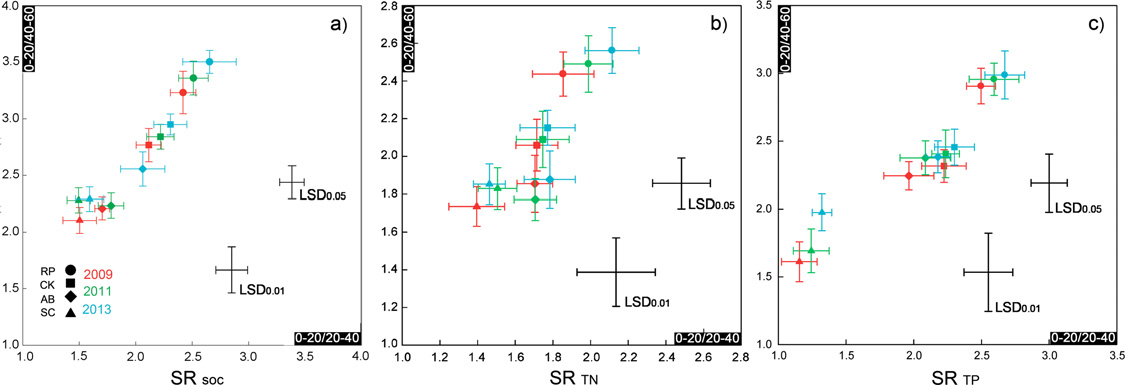
Stratification ratios of SOC, TN, and TP of different land use types and soil depth in 2009, 2011, and 2013. Abscissa and ordinate value is the stratification ratios of each symbol in 0–20:20–40cm and 0–20:40–60cm, respectively. Symbols with different shape represent different land use types: roundness is RP, square is CK, rhomb is AB and triangle is SC. Symbol with different color represent different experiment year: red for 1999, green for 2011 and blue for 2013. Bars across the symbol represent standard errors. Cross LSD shows its magnitude (at P < 0.05 or 0.01) in horizontal and vertical to compare means for stratification ratios of different land use types and years in 0–20:20–40cm and 0–20:40–60cm.

The SR values for the SOC, TN and TP contents at depths of 0–20:20–40 and 0–20:40–60 were affected by the land use types (RP, CK, AB and SC) and the years of vegetation restoration, as shown in Fig 6. The SR values were mostly >2 in the re-vegetated lands and <2 in the SC, which indicates considerable improvement in the soil quality by converting cropland to vegetation land. The SR for the SOC of RP, CK, AB and SC significantly increased by 5.9%-21.1% in 0–20:20–40 and 6.6%-15.8% in 0–20:40–60 from 2009 to 2013 (P<0.01). The largest increment appeared in AB, and the lowest appeared in the SC (0–20:20–40 cm) and CK (0–20:40–60 cm). The SRs for the TN of the four land use types showed insignificant increasing trends from 2009 to 2013 in both the 0–20:20–40 and 0–20:40–60 layers, except for RP in the 0–20:20–40 layer, which was significant (P<0.05). Additionally, the SRs for the TP of re-vegetated land types also showed insignificant increasing trends from 2009 to 2013 in both the 0–20:20–40 and 0–20:40–60 layer, but the TP SR in the SC increased significantly (P<0.01).

The SRs for the SOC, TN, and TP contents in 2013 were analysed in detail to reveal the differences among the different land use types (Fig 6). The SRs for the SOC contents of RP, CK and AB were significantly higher than that in the SC, with differences of 1.1, 0.7 and 0.5 in the 0–20:20–40 layer and 1.2, 0.7 and 0.3 in the 0–20:40–60 layer, respectively (P<0.01). The SRs for the TN contents of RP, CK and AB were significantly higher than that in the SC in the 0–20:20–40 layer, which were ordered RP>CK>AB>SC. Additionally, the SRs for the TN contents of RP and CK were significantly higher than that in the SC in the 0–20:40–60 layer, but no significant difference existed between AB and SC (P<0.05). The SRs of the TP contents in RP, CK and AB were significantly higher than that in the SC for both the 0–20:20–40 and 0–20:40–60 layers, but no significant difference existed between CK and AB (P<0.05). Thus the differences in the SRs for the SOC, TN, and TP contents of RP in the 0–20:20–40 and 0–20:40–60 layers were significantly higher than those among other land use types (P<0.01), which were ordered RP>CK>AB>SC in each year. The SR distributions of the SOC, TN and TP contents among the different land use types and depths in 2009 and 2011 were consistent with those in 2013.

**Fig 7:**
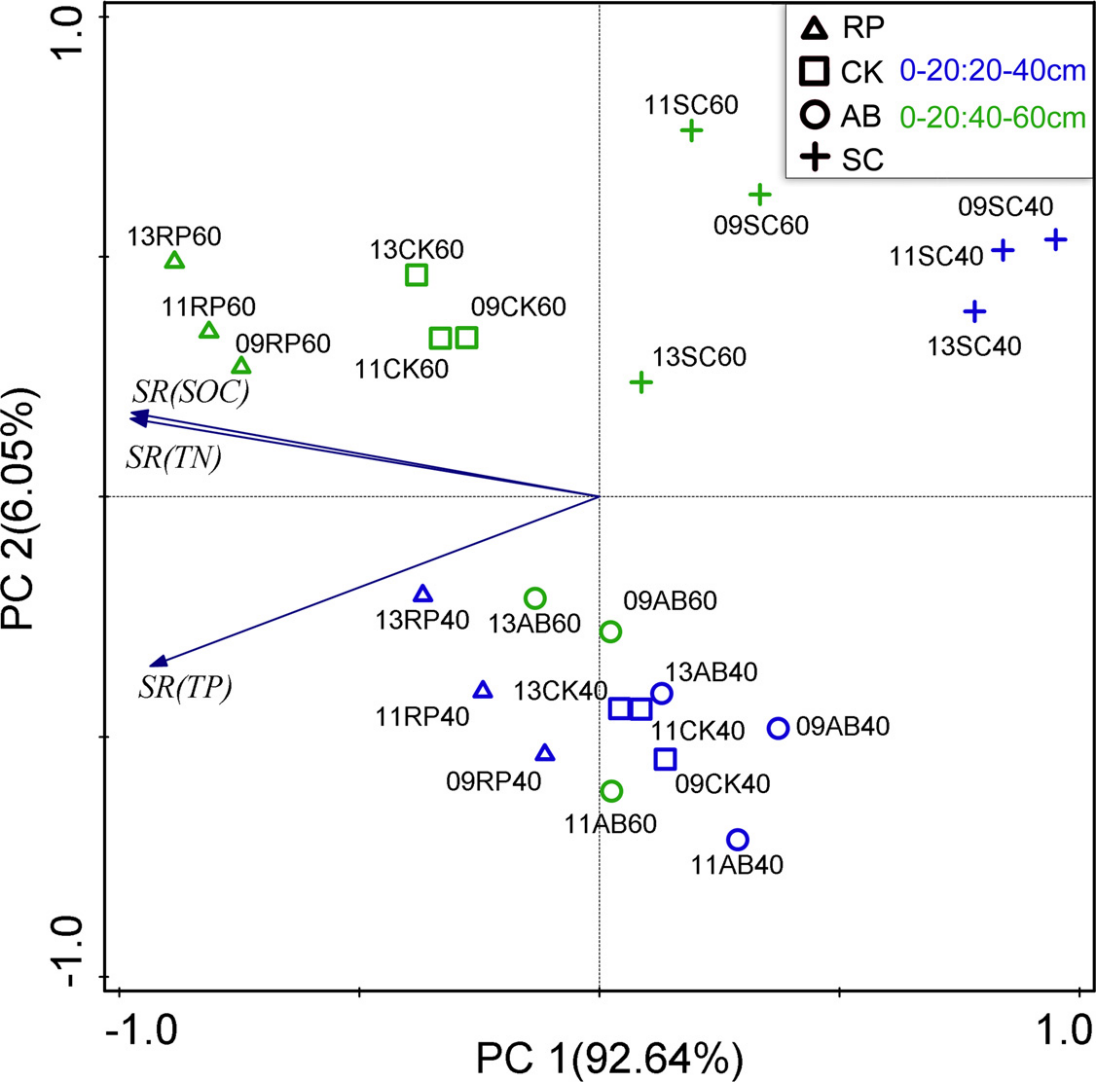
Principal component analysis (PCA) loadings for SR of soil SOC, TN and TP samples. Symbols with different shape represent different land use types: triangle is RP, square is CK, roundness is AB and cross is SC. Symbol with different color represent different soil depth: blue for 0–20:20–40cm and green for 0–20: 40–60cm.

The PCA loadings for the SRs of the soil SOC, TN and TP samples are shown in Fig 7. The PC1 and PC2 successfully differentiated the samples, and the cumulative variance contributions reached 99.2% (PC1 explained 92.6% and PC2 explained 6.1%). All the samples were roughly grouped by the land use type and soil depth in the factor loading plots. The years seemingly did not group the sample plots apparently in Fig 6.

The ANOSIM R-values of the SR values of SOC, TN and TP are shown in Table 4. The results showed that the SRs of SOC, TN and TP were all dissimilar from the groups that were derived from each treatment, except for the restoration years for the SRs of TN and TP (P<0.01). The most influential factor toward the SOC and TN storages was the soil depth (R = 0.998 and 0.769, P<0.01), and that toward the TP storages was the land use type (R = 0.0.837, P<0.01). These results are inconsistent with the storages results in Fig 4. Taken together, the soil depth was the most influential factor for the SRs of SOC, TN and TP, with land use type the second-most influential.

**Table 4:** R-value for ANOSIM of SRs of SOC, TN and TP^a^.

	SR^b^	SR(SOC)	SR(TN)	SR(TP)
**Land use type**	0.971	0.949	0.709	0.837
**Year**	0.379	0.588	0.083NS	0.143NS
**Soil depth**	0.997	0.998	0.769	0.617

^a^ The R-values are the average value of pairwise tests for each treatments and are all statistically significant except for those labeled with “NS”.

^b^ The R-values for “SR” are from the comprehensive analysis of each treatment for SRs of SOC, TN and TP

### Relationship between storage and SR of SOC, TN and TP

The correlations among the storages and SRs of the SOC, TN and TP contents among the different land use types were analysed (Table 5). Significant positive correlations existed between SOC and TN storage at depths of 20–40 cm and 40–60 cm. Positive correlations also existed between the SRs for the SOC and TN in the corresponding soil layer (0–20:20–40 cm, 0–20:40–60 cm) in each year (P<0.05). Meanwhile, the correlative coefficients of SOC and TN decreased as the soil depth increased. However, no consistently significant correlation between the storage and SR of TP was found.

**Table 5:** Correlations between storage and SR of SOC, TN and TP. Cor. (Sig.) is short for correlation coefficient (significance). **represent significant at P<0.01 level; * represent significant at P<0.05 level; NS represent no significant correlation.

Year	Soil Depth	SOC	TN	TP
	(cm)	Cor.(Sig.)	Cor.(Sig.)	Cor.(Sig.)
**2009**	20–40	0.993 **	0.930 *	-0.610 NS
	40–60	0.976*	0.830 *	0.387 NS
**2011**	20–40	0.986 **	0.943 *	-0.401 NS
	40–60	0.941*	0.714 NS	0.613 NS
**2013**	20–40	0.944 *	0.909 *	-0.358 NS
	40–60	0.962 *	0.885 *	0.682 *

## Discussion

### SOC, TN, and TP contents and storages following vegetation recovery

Land use changes significantly influence the circulation of soil nutrients[38]. The present research demonstrated that the contents and storages of SOC, TN and TP in re-vegetated lands were significantly higher than those of cropland, indicating the accumulation of C, N and P when converting cultivated land into vegetation. These results are consistent with previous studies [10,15,39]. This appearance was arising from the increasing of plant biomass after re-vegetation, which cause more organic litter to return to the soil [40,41]. Additionally, serious water and soil run-off result in grievous nutrient loss in the LPC, which was considered as another reason for the low soil fertilizer attributes in cropland[27]cropland.

The accumulation of soil C, N and P is affected by multifarious factors [42]. The ANOSIM results showed that the land use types influences the SOC, TN and TP storages the greatest, with the soil depth and restoration years the second- and third-most influential factors, respectively. The different land use types had different potentials to affect the soil C, N and P, which was consistent with Qiu et al. [43]. These differences in nutrient accumulation ability may derive from several reasons. First, the processes of nutrient return were different. The increasing SOC and TN in abandoned grasslands mainly originated from withered grass that returned to the soil but were jointly influenced by understory herbaceous plants and litter falls from trees in afforestation (shrubs and forests) [27]. Secondly, the understory species and species diversity were different among different re-vegetated lands. In addition, the specific function like nitrogen-fixing capacity may be another important factor favoring SOC and TN sequestration [44]. Unlike the SOC and TN, the TP content and storage did not exhibit significant increase from cropland to vegetation restoration land in the subsoil as it may because that phosphorus transformations in soil ecosystems are mainly driven by biochemical mineralization rather than biological factors[45].

The contents and storages of the SOC, TN and TP were observed to be vertically stratified with soil depth because of direct disturbances from the environment and nutrient return (such as litter and roots) on the surface soil. The SOC and TN in the subsoil layers significantly increased from the SC to re-vegetated land, although this increase was less than that in the top layer. This result is consistent with the studies of Zhao et al. [20] and Fu et al.[5] in the Loess Hilly Region. The result also showed that approximately 50% of the SOC, TN and TP from the 0–60 cm soil profile were stored in the subsoil. This percentage is close to Batjes [46] and Jobbágy’s [47]results, which showed that more than 50% of the global SOC is stored in subsoil from 30 to 100 cm and confirms that the subsoil layer is indispensable in assessing the accumulation of soil nutrients.

The present study indicated that the contents and storages of SOC, TN and TP in re-vegetated land over the long-term (30 years) were still significantly increasing with the number of restoration years. These results were not completely consistent with those from Wang et al.’s [27] study, who concluded that the soil SOC content in artificial forests may peak after 17–18 years in the semi-arid Loess Plateau. Because the climatic and edaphic conditions and management measures in Wang et al ‘s study were exactly the same as those in the present study, we speculate that this difference may be attributed to differences in the former land use, which Navarro [48] and Paul et al. [49] reported could influence the soil dynamics 30 years after reforestation.

### Using the stratification ratios of the SOC, TN and TP contents to evaluate soil quality

In this study, the SRs of SOC, TN and TP in the 0–20:20–40 and 0–20:40–60 layers significantly varied among different land use types (Fig 6). These results were consistent with those by Wang et al. [50] and Zhao et al.[20], who showed significantly different SRs among different land use types in the LPC. However, the SR values in the present study were generally higher than those in Wang et al.’s [50] study, with a ratio of 1.14–1.85 for SOC. This divergence may be due to differences in the calculation method (the topsoil layer was defined as 0–5 cm in [50]), meteorological distinctions (e.g., precipitation was 535.0 mm in their research area and 505.0 mm in this study) and the soil conditions. Franzluebbers [16] stated that the SRs of SOC and TN were >2, which indicated an improving soil quality. Our results showed that the SR values of re-vegetated land were mostly >2 for the SOC, TN and TP contents in the subsoil. In contrast, the SR in the cropland were mostly <2 for the 0–20:20–40 cm and 0–20:40–60 cm depth ratios. Similar results were also reported in Zhao et al.’s study [20]. These observations indicate that the soil quality in the LPC could be improved by converting cultivated land to vegetation and that vegetation restoration was beneficial for the accumulation of surface soil SOC, TN and TP. Moreover, topsoil layers prevent water and soil erosion, which is crucial in the Loess Plateau [51]. Thus, a higher SR value indicates a better capacity for water-soil conservation.

Additionally, a majority of SR values for TN and TP from 2009 to 2013 and for SOC, TN and TP between neighbouring observation years (2009 and 2011, 2011 and 2013) showed a persistently increasing but insignificant trend, which revealed that the SRs for the soil SOC, TN and TP in artificially vegetated land should be assessed over long time scales. The continuously increasing SRs also indicate that the SOC, TN and TP had not yet reached saturation after 30 years of restoration.

The relationship between the storage and SR values indicated that the SOC and TN storage were significantly positively correlated to the SR in the corresponding soil depth. This finding was partly similar to Sá and Lal’s study [18], which showed that the SR of SOC in cultivated land was positively correlated with the amount of sequestered SOC. Higher SRs can reflect good soil nutrient accumulation in the topsoil, which is important for soil restoration, soil erosion control and water infiltration[16]. On the other hand, a close relationship between SOC and TN storages and SRs reveals that the SR can indicate the level of storage in deeper soil; the storage of soil nutrients is usually used as the indicator of soil quality [4]. Therefore, SRs are recommended as a good indicator to assess the soil quality in artificial vegetation.

## Conclusions

Artificial vegetation can increase the contents and storages of SOC, TN and TP, in both topsoil (0–20 cm) and subsoil (20–60 cm), compared to cropland in LPC over 30 years. Significantly higher SRs of SOC, TN and TP were also observed in re-vegetated land than in cropland and were mostly >2. The largest influential factors for the SOC, TN and TP storages and SRs were the land use types and soil depth. A significant positive relationship existed between storages and SRs. These findings conclude that (1) converting cropland to vegetation significantly enhanced the accumulation of SOC, TN and TP in both topsoil and subsoil; (2) SRs are a practicable indicator to assess the soil quality in artificial vegetation; and (3) the soil quality was improved by converting cropland to vegetation in the LPC. This study underscores the necessity of including subsoil when evaluating soil nutrition accumulation and proposes that SRs can be used as an indicator of the soil quality in artificial vegetation.

## Supporting Information

S1 Table The detailed ANOVA results table. (XLSX)
